# Crystal Structures of Wzb of *Escherichia coli* and CpsB of *Streptococcus pneumoniae*, Representatives of Two Families of Tyrosine Phosphatases that Regulate Capsule Assembly

**DOI:** 10.1016/j.jmb.2009.07.026

**Published:** 2009-09-25

**Authors:** Gregor Hagelueken, Hexian Huang, Iain L. Mainprize, Chris Whitfield, James H. Naismith

**Affiliations:** 1 Centre for Biomolecular Sciences, The University of St. Andrews, Fife KY16 9RH, UK; 2 Department of Molecular and Cellular Biology, University of Guelph, Guelph, Ontario, Canada N1G 2W1

**Keywords:** CPS, capsular polysaccharide, PHP, polymerase and histidinol phosphatase, pNPP, *para*-nitrophenylphosphate, enzyme mechanism, kinase, phosphatase, pathogenic bacteria, capsule

## Abstract

Many Gram-positive and Gram-negative bacteria utilize polysaccharide surface layers called capsules to evade the immune system; consequently, the synthesis and export of the capsule are a potential therapeutic target. In *Escherichia coli* K-30, the integral membrane tyrosine autokinase Wzc and the cognate phosphatase Wzb have been shown to be key for both synthesis and assembly of capsular polysaccharides. In the Gram-positive bacterium *Streptococcus pneumoniae*, the CpsCD complex is analogous to Wzc and the phosphatase CpsB is the corresponding cognate phosphatase. The phosphatases are known to dephosphorylate their corresponding autokinases, yet despite their functional equivalence, they share no sequence homology. We present the structure of Wzb in complex with phosphate and high-resolution structures of apo-CpsB and a phosphate-complexed CpsB. We show that both proteins are active toward Wzc and thereby demonstrate that CpsB is not specific for CpsCD. CpsB is a novel enzyme and represents the first solved structure of a tyrosine phosphatase from a Gram-positive bacterium. Wzb and CpsB have completely different structures, suggesting that they must operate by very different mechanisms. Although the mechanism of Wzb can be inferred from previous studies, CpsB appears to have a tyrosine phosphatase mechanism not observed before. We propose a chemical mechanism for CpsB based on site-directed mutagenesis and structural data.

## Introduction

Bacterial capsules (capsular polysaccharides or CPSs) play critical roles in interactions between bacteria and their environments. They are often the point of first contact with the host and represent important virulence factors for both Gram-negative and Gram-positive pathogens. CPSs are frequently associated with protection of bacteria against the host immune system.^1^ Several distinct processes for CPS synthesis and assembly have been described. One of these, known as the Wzy-dependent pathway, is found in many Gram-positive and Gram-negative bacteria.^1,2^ In the Wzy-dependent process, lipid-linked intermediates carrying individual repeat-units of the CPS are exported across the cytoplasmic membrane, where they form substrates for the polymerization step, which requires the characteristic oligosaccharide polymerase Wzy. Full polymerization activity requires the involvement of a member of the polysaccharide co-polymerase family; examples include Wzc in *Escherichia coli* and CpsCD in *Streptococcus pneumoniae*.^2,3^ Wzc and CpsCD are also members of the tyrosine autokinase family (BY kinases).^3,4^ Wzc is an integral membrane protein consisting of two transmembrane helices flanking a periplasmic domain and a cytosolic C-terminal region containing the tyrosine kinase and an extended tyrosine-rich tail (reviewed by Cuthbertson *et al*. [2]). Mutation of the Walker box motif in Wzc abolishes both phosphorylation and capsule assembly. The *wzb* gene has been shown to be essential for CPS expression,^5^ and the extent of phosphorylation influences the amount of CPS produced.^6^ Wzb is a member of the cysteine-dependent tyrosine phosphatase superfamily and, given its location in the genome, is assumed to target Wzc. Thus, at least for *E. coli* K-30, the cycling of phosphorylation appears to be crucial to the proper functioning of the Wzc-dependent system. In the Gram-positive *S. pneumoniae*, the protein complex CpsCD is thought to mimic Wzc, but it utilizes separate polypeptides for the transmembrane/periplasmic kinase (CpsC) and the cytoplasmic kinase (CpsD) domains.^2,3^ The phosphorylation state of CpsCD also influences both the amount of CPS formed^7–9^ and the extent of CPS attachment to the cell wall.^10^ While there is evidence that phosphorylation is not universally required for CPS assembly, it is likely to play an important role in a number of diverse but clinically important bacterial systems.

The structure of the cytosolic tyrosine autokinase domain from a close homolog of Wzc (called Etk) has been reported recently.^11^ Furthermore, the structure of the functional kinase domain of the CpsCD homolog CapAB from *Staphylococcus aureus* has been determined.^12^ The structures reveal close similarities to the Mrp–MinD subfamily of P-loop ATPases.^13^ Dephosphorylation of the tyrosine cluster at the C-terminus of the CpsAB kinase leads to oligomerization of the kinase domain into octamers, but it is not yet known what effect this has on the full-length protein *in vivo*.^12^

The precise mechanism by which phosphorylation influences biosynthesis of CPS remains to be defined, and it is unclear how the information contained in the C-terminal tyrosine cluster is structurally translated into a regulation of the whole assembly system. Its involvement may not be confined to a single step since both the kinase and the phosphatase could modify other proteins participating in sugar nucleotide precursor biosynthesis^14–16^ or initiating glycosyltransferases.^17^ Following biosynthesis, the assembly processes diverge in Gram-positive and Gram-negative bacteria because the latter must export the CPS across the periplasm and outer membrane. This process also involves the polysaccharide co-polymerase protein Wzc, whose periplasmic domain interacts with a novel outer membrane protein, Wza,^18,19^ belonging to the OPX (outer membrane polysaccharide export) family.^2,20^

Despite the functional parallels, the Gram-negative and Gram-positive phosphatases, such as Wzb and CpsB, are completely unrelated at the sequence level. Wzb is a homolog of low-molecular-weight protein-tyrosine phosphatases, which occur in both prokaryotes and eukaryotes, and these enzymes characteristically function via a conserved cysteine residue that serves as nucleophile.^21^ Our understanding of this enzyme family is supported by biochemical and structural studies on the eukaryotic family members^21–23^ and, for prokaryotic representatives, by the NMR structure of uncomplexed Wzb.^24,25^ In contrast, CpsB and related phosphatases belong to the polymerase and histidinol phosphatase (PHP) family,^26^ and biochemical studies indicated that CpsB phosphatases are metal-dependent enzymes.^27–29^ No structural information for prokaryotic CpsB-like phosphatases is available to date. Since the dephosphorylation of bacterial tyrosine kinases, such as Wzc and CpsCD, is a crucial part in the capsular assembly process in both Gram-negative and Gram-positive bacteria, the phosphatases represent potential targets for drug development efforts. We have thus characterized representatives from each family: Wzb from *E. coli* K-30 (denoted Wzb_K-30_) and CpsB from *S. pneumoniae* TIGR4 (denoted Cps4B).

We present here a 2.5-Å resolution structure of a Wzb_K-30_–PO_4_^3−^ product complex, a 2.2-Å structure of Wzb_K-30_ in complex with sulfate, the 1.9-Å apo-structure of Cps4B and the 2.2-Å Cps4B phosphate product complex. Comparison of the structures, coupled to site-directed mutagenesis, yields important mechanistic insights into these enzymes. Furthermore, we show that both Wzb and Cps4B are capable of completely dephosphorylating full-length Wzc, establishing that Cps4B activity is not confined to its cognate CpsCD complex.

## Results

### Structure of the Wzb_K-30_ product complex

We solved two structures of Wzb_K-30_—one in complex with the phosphate reaction product (2.5 Å resolution) and another in complex with sulfate, a mimic of the reaction product (2.2 Å resolution). The two structures superimpose with an rmsd of 0.3 Å, and although the phosphate-complexed structure is partially disordered, the active site is well defined and the positions of the two ligands are identical.

**Fig. 1:**
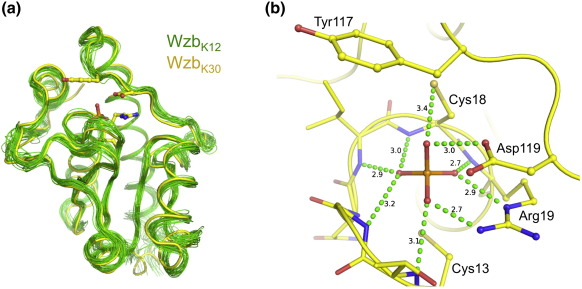
(a) Comparison of the Wzb_K-30_ product complex structure (yellow) with the NMR structure of apo-Wzb_K-12_ (green; PDB ID 2FEK). The phosphate ion that is bound to the active site of the crystal structure and selected active-site residues are shown as ball-and-stick models. (b) Close-up view of the active site. Numbers in black are distances in angstrom. Selected interactions are indicated by dashed lines. Numbers in black are distance in angstrom.

In terms of tertiary structure, Wzb_K-30_ is essentially identical with the NMR structure of apo-Wzb_K-12_, with an rmsd of 0.8 Å for 132 superposable C^α^ atoms (Fig. 1a). Briefly, the enzyme has a mixed αβ structure with a central four-stranded parallel β-sheet surrounded by seven α-helices. The active-site cavity is marked by the reactive Cys13 residue, which sits on top of the β-sheet. The Wzb_K-30_ structure is a product complex, containing a phosphate ion that is coordinated by residues Arg19 (N, N1, N2), Gly15 (N), Asn16 (N), Ile17 (N), Cys18 (N) and Asp119 (O2). The phosphate is positioned such that the phosphorus oxygen bond is in line with the S atom of Cys13 (Fig. 1b), correctly oriented for S_N_2 attack. Previous biochemical data have established that a Cys13Ser mutation eliminates phosphatase activity,^5^ and the structure confirms that Cys13 is indeed the catalytic nucleophile. NMR-based phosphate titrations have shown that the binding of phosphate to the active site leads to structural rearrangements.^24,25^ A superposition of the ligand-free NMR structure of Wzb_K-12_ onto our Wzb_K-30_–PO_4_^3−^ complex structure shows that phosphate binding leads to an ordering not only of the residues that are directly bound to the phosphate but also of the nucleophilic Cys13 and Tyr117, which are adjacent to phosphate ligands and believed to be important for substrate binding (Fig. 1b; Fig. S1).^24^

### Cps4B is a novel phosphatase with three active-site metals

**Fig. 2:**
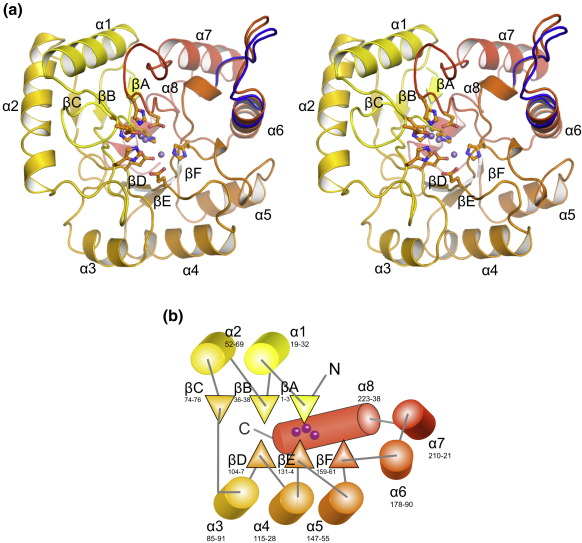
(a) Crystal structure of Cps4B (stereo pair). The structure is shown in ribbon representation and colored with a gradient running from yellow (N-terminus) to red (C-terminus). The metal ions bound to the active site are shown as purple spheres; metal ligands, in ball-and-stick mode. The alternative conformation of the flexible loop connecting α6/βF is shown in blue. (b) Topology plot of Cps4B. Helices are shown as cylinders; strands, as arrows; and loops, as gray lines. The approximate positions of the metal ions, with respect to the secondary structure elements, are indicated by purple spheres.

We solved the structure of Cps4B in two crystal forms, *P*2_1_2_1_2_1_ and *P*4_3_2_1_2. Both structures are identical with the exception of loop 169–177, which shows a significant conformational change between the two crystal forms, indicating a degree of flexibility in this part of the structure (Fig. 2a). The loop is involved in crystal contacts in both structures, but it would protrude into the surrounding solvent if the protein was in solution.

The structure reveals that Cps4B is a member of the PHP-fold family^26^ and consists of two three-stranded parallel β-sheets with a ring of α-helices on the outside of the structure. Three metal ions are bound adjacent to one another between the β-sheets (Fig. 2). The fold can be described as a distorted TIM barrel.^26^ The SSM algorithm^30^ was applied to search the protein structure database for similar structures, and the proteins identified with the most similarity were two phosphotriesterases from *E. coli* [PHP, Protein Data Bank (PDB) ID 1BF6]^31^ and *Sulfolobus solfataricus* (SsoPox, PDB ID 2VC5).^32^ These structures are representatives of the PHP fold; the structural similarity is relatively low, with rmsd values larger than 2.6 Å and *Z*-scores lower than 5.0 for both structures (Fig. S2). Chemically, there is a link between the reactions catalyzed between Cps4B and phosphotriesterases, such as PHP and SsoPox, in that these enzymes both hydrolyze phosphate ester bonds, although the phosphates are attached to very different organic groups. Furthermore, phosphotriesterases use only two metal ions and have different residues (including a carboxylated lysine) involved in chelating the metal ions.^31–33^

**Fig. 3:**
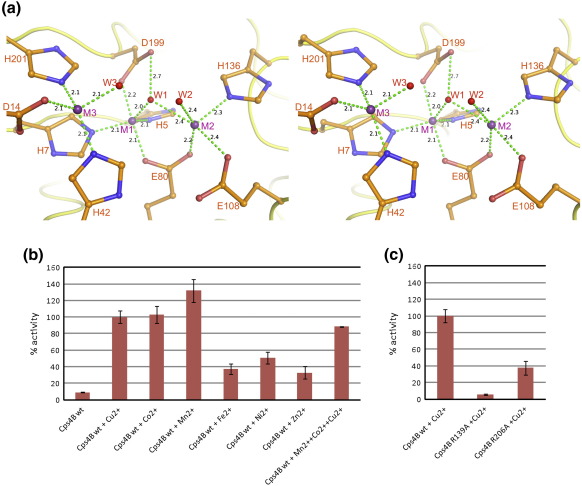
(a) Metal binding site of Cps4B (stereo pair). Residues that interact with metal M1, M2 or M3 (purple spheres) are shown in ball-and-stick mode. Water ligands W1, W2 and W3 are depicted as red spheres. Selected interactions are shown as dashed lines. Numbers in black are distances in angstrom. (b) Activity of Cps4B in the presence of different divalent metal ions. The activity of Cps4B in the presence of Cu^2+^ was arbitrarily set to 100%. Error bars indicate the standard deviation of measurements (quadruplicates). (c) Activity of wild-type Cps4B compared with that of mutants Cps4B_R139A_ and Cps4B_R206A_. Error bars indicate the standard deviation of measurements (quadruplicates).

Cps4B co-purifies with three metal ions bound to the active site (Fig. 3a). Metal M1 is coordinated in a trigonal-bipyramidal fashion by two axial ligands (Glu80 and Asp199) and three equatorial ligands, His5, His7 and W1. Since water molecule W1 also serves as a ligand for metal M2, it is most likely deprotonated and thus represents a shared hydroxyl ion. M2 is coordinated by Glu80, Glu108, His136 and two water molecules, W1 and W2, in a slightly distorted square-based pyramidal ligand shell. Glu80 serves as a bidentate ligand for metals M1 and M2. The distance between M1 and M2 is 3.5 Å, which is typical for a binuclear metal centre.^34^ Metal M3 has distances of 4.7 and 5.6 Å to metals M1 and M2, respectively, and has a tetragonal ligand sphere composed of His42, Asp14, His201 and water molecule W3.

The anomalous peak for M3 is significantly smaller than peaks corresponding to M1 and M2, indicating that a different metal is bound to this site (Fig. S3). Although the purified enzyme is loaded with metal ions, it is a relatively poor catalyst with artificial substrate *para*-nitrophenylphosphate (pNPP). As previously described for homologs of Cps4B,^27–29^ we found that the addition of Cu^2+^, Co^2+^ and Mn^2+^ ions to the reaction increases the activity of Cps4B by more than 10-fold (Fig. 3b). Fe^2+^, Ni^2+^ and Zn^2+^ lead to a much lower activation of the enzyme, and since these are most abundant in expression media and purification buffers, the low initial enzyme activity is potentially due to incorrect metal ion loading.

### The Cps4B–PO_4_^3−^ complex

**Fig. 4:**
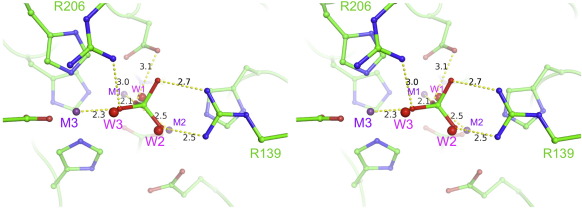
Binding of phosphate to the active site of Cps4B (stereo pair). The phosphate and selected residues are shown in ball-and-stick mode. The metals are indicated as purple spheres, whereas metal-bound water molecules from the apo-structure that are replaced by the phosphate are shown as red spheres. The positions of catalytically important residues Arg139 and Arg206 are indicated. Selected interactions are shown as yellow dashed lines, and distances are given in angstrom (black numbers).

To compare the mechanisms of Cps4B- and Wzb-like enzymes, we solved the structures of Cps4B in complex with a phosphate ion. The apo- and phosphate-bound structures of Cps4B are almost identical, with an rmsd of 0.14 Å. The phosphate is tightly bound to the active site by a multitude of interactions involving all three metal ions and amino acid residues Arg139 and Arg206. Interestingly, the phosphate replaces all three metal-bound solvent molecules (W1, W2 and W3; Figs. 3a and 4). Also, due to their interaction with the ligand, both Arg139 and Arg206 (which are partly disordered in the apo-structure) are much better defined in terms of electron density.

**Fig. 5:**
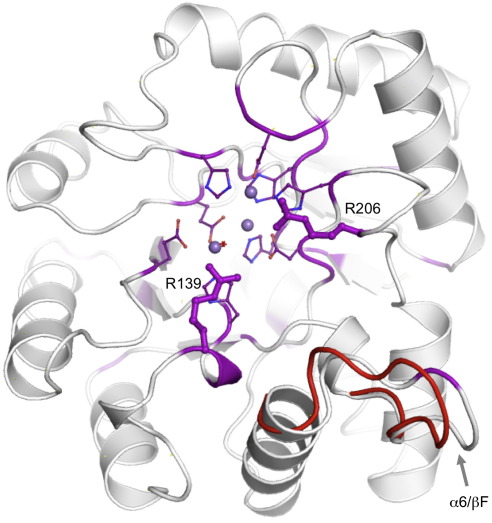
An alignment of Cps4B with similar phosphatases from polysaccharide biosynthesis systems (Fig. S4) was mapped onto the Cps4B structure. Conserved residues are shown in purple. Catalytically important residues Arg139 and Arg206 are labeled. The alternative conformation of loop α6/βF is shown in red.

To identify residues that might play a role in substrate binding and catalysis, we mapped an alignment^27^ of Cps4B homologs onto the Cps4B structure (Fig. 5; Fig. S4). This procedure reveals that both phosphate ligands Arg139 and Arg206 are conserved and therefore most likely important for Cps4B-mediated catalysis. In order to test this hypothesis, we prepared Cps4B mutants Arg139Ala and Arg206Ala (R139A, R206A) and checked both for catalytic activity. Whereas Cps4B_R206A_ shows approximately 40% remaining activity, Cps4B_R139A_ has only 5% of wild-type activity (Fig. 3b). Some of the active-site metal ligands (e.g., His5, His7, His42 and His201; Cps4B numbering) and residues interacting with metal ligands (Asp3; Cps4B numbering) have been mutated in other studies, and the corresponding mutants showed 1%–20% residual activity.^27–29^ Our structure shows that these residues are clearly important for the stability of the metal cluster and thus for the function of the enzyme. Nevertheless, their role in catalysis is indirect (see below), and we therefore identify Arg139 as the catalytic key player of Cps4B.

Additionally, our structure reveals that some of the activity-affecting residues that were mutated by LaPointe *et al*. (down to ∼ 5% of wild-type activity^27^) (D64N, R68A, R69A, C78S; numbering of LaPointe *et al*.) are situated on the molecular surface or distant from the active site of Cps4B and thus most likely not involved in catalysis. However, it should be noted that the corresponding experiments were performed at elevated temperatures (47 or 75 °C) and that the observed loss in activity might be due to a destabilizing effect of the mutations under those conditions.

### Cps4B is able to dephosphorylate Wzc_K-30_ and is thus not specific to CpsCD

**Fig. 6:**
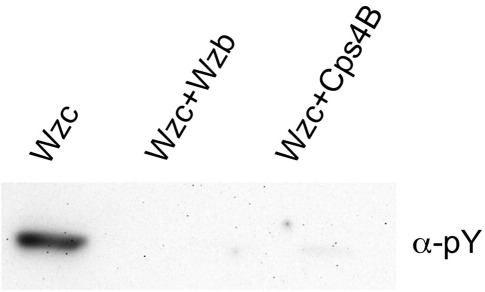
Western blot analysis (anti-phosphotyrosine antibody) showing that both Wzb_K-30_ and Cps4B are capable of dephosphorylating Wzc_K-30_.

Full-length Wzc_K-30_ was purified and extracted from *E. coli* harboring the *wzc*_K-30_ gene on an inducible plasmid. Western immunoblot analysis with an anti-pY antibody indicates that the isolated protein is phosphorylated, as previously reported.^5,6^ When purified Wzb_K-30_ or Cps4B was added to Wzc_K-30_, the kinase was completely dephosphorylated (Fig. 6), establishing that both Wzb_K-30_ and Cps4B recognize and dephosphorylate the tyrosine cluster of Wzc_K-30_.

## Discussion

### Catalytic mechanism of Cps4B

The mode of binding of phosphate in the active site of Cps4B suggests a possible mechanism (Fig. 4). Many metal-dependent phosphatases employ a metal-bound water molecule or hydroxyl ion as nucleophile.^36^ The phosphate ion bound to the active site of Cps4B replaces all three metal-bound water molecules W1, W2 and W3. Our structural data give no evidence for another residue, or group, that could act as nucleophile. The Cps4B–PO_4_^3−^ structure most likely resembles a post-reaction state where the phosphate group has been transferred from tyrosine to the nucleophilic water molecule. Considering steric restraints (the leaving group is a large protein) and the fact that most phosphoryl-transfer reactions proceed via an in-line nucleophilic attack,^37^ only W1 remains a plausible candidate as the nucleophile of Cps4B. Notably, the shared hydroxyl W1 is also proposed to be the nucleophile in the structurally related phosphotriesterases.^32,33^ Phosphotriesterases break down otherwise toxic organophosphorus triesters by nucleophilic attack of a metal-activated hydroxyl at the phosphorus atoms.^32,33^ We propose that in Cps4B, the incoming phosphotyrosine binds to the active site via Arg139, Arg206, M2 and M3. The strong ionic interaction of the phosphate group with Arg139 helps position the phosphorus atom directly over the nucleophile, W1 (Fig. 4), and additionally displaces negative charge from the phosphorus atom, making it more amenable for the subsequent in-line nucleophilic attack by W1. During the S_N_2 displacement, the positively charged elements surrounding the active site (Arg139, Arg206, M1, M2 and M3) will help stabilize the developing negative charge of the trigonal-bipyramidal intermediate. Additionally, after cleavage of the phosphoester bond, this positive potential should stabilize the resulting tyrosinate by lowering the p*K*_a_ of the phenolic OH group. The tyrosinate would finally be protonated by solvent as it leaves the active site. A similar stabilization of a tyrosinate by a positive charge has been described for SDR dehydrogenases.^38^ In broad outline, the chemistry is similar to that of the phosphotriesterases.^32,33^

### Comparison of the active sites of Cps4B and Wzb

The catalytic mechanism of cysteine-dependent low-molecular-weight protein-tyrosine phosphatases, such as Wzb_K-30_, is well established.^21^ Briefly, the phosphotyrosine substrate is bound by a conserved positively charged arginine residue (Arg19 in Wzb_K-30_) and a ring of backbone amides that present a positively charged pocket. The reaction would proceed in two distinct steps—S_N_2 displacement of tyrosine by the thiol (Cys13 in Wzb_K-30_) (Fig. 1b) that is followed by protonation of the tyrosinate through either Asp119 or a second conserved cysteine (Cys18; Fig. 1b).^21,39,40^ This creates an enzyme-bound thiophosphate ester intermediate and free tyrosine. In the second step, the thiophosphate ester is hydrolyzed by an incoming water molecule (activated by Asp119) in another S_N_2 displacement, this time generating free phosphate and regenerating the thiol.

**Fig. 7:**
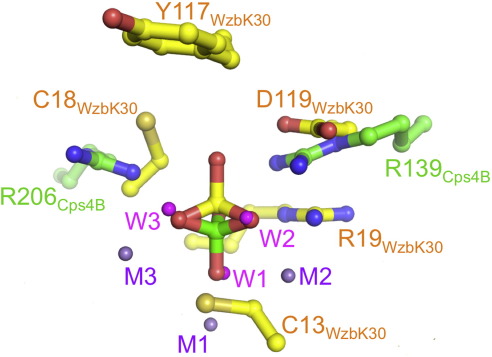
Product-based alignment of the Wzb_K-30_ (yellow) and Cps4B (green) structures. Metals M1, M2 and M3 of Cps4B are depicted as purple spheres. Metal-bound water molecules of Cps4B (W1, W2 and W3) are shown as magenta spheres.

Although the structures of Wzb_K-30_ and Cps4B are completely different and evolutionary unrelated, the fact that both act on the same substrate prompted us to look for common chemical features in the active sites of both enzymes. We superimposed our product complexes of Wzb and Cps4B by matching three of the phospho-oxygen atoms (Fig. 7). Since the phosphate ion in the Wzb structure presents a pre-reaction state and the phosphate ion in the Cps4B structure presents a post-reaction state, we superposed the phosphate of Wzb onto the Cps4B phosphate such that it mimics the substrate state (before the nucleophilic hydroxyl attacks and inverts the geometry). This “product-based alignment” reveals that metal ions M2 and M3 serve in part as a structural counterpart of the ring of backbone amides (a positive charge) that position the phosphate group in Wzb_K-30_ (Figs. 1b and 7). The C3 symmetry of the phosphate group along the reaction axis allows three ways of superposing the two phosphate groups. For one of the three possible choices, we found a good structural alignment of the catalytically important arginine residues Arg19 of Wzb and Arg139 of Cps4B (Fig. 7). The nucleophilic Cys13 of Wzb_K-30_ superposes with the shared hydroxyl, W1, which is bound between M1 and M2 of Cps4B. This observation supports our assumption that W1 provides the nucleophile in the Cps4B-catalyzed dephosphorylation reaction. As Cps4B does not create an enzyme-bound intermediate, it does not need a chemical counterpart for the conserved Asp119 of Wzb_K-30_.^21,40^

### Substrate binding and specificity

In Wzb_K-30_, a conserved tyrosine residue (Tyr117 of Wzb_K-30_) is believed to be important for binding of the phosphotyrosine at the active site.^24^ Modeling a phosphotyrosine onto the bound phosphate in our Wzb-PO_4_^3−^ complex structure corroborates this proposal since the modeled tyrosine forms a stacking interaction with Tyr117 (Fig. S5). The substrate-based alignment (see above and Fig. 7) reveals that Cps4B does not possess an obvious counterpart for this tyrosine.

As mentioned above, loop βF/α6 shows a significant conformational change between the two crystal forms *P*2_1_2_1_2_1_ and *P*4_3_2_1_2 (Fig. 2a). It is close to the active site and contains several hydrophobic amino acids that could potentially form hydrophobic interactions with the incoming substrate (e.g., Leu172, Phe173 and Tyr177). The loop is not well conserved over its full length (Fig. 5 and Fig. S4), but many Cps4B homologs contain a FG(D/E)K/R motif in this position, where the phenylalanine corresponds to Phe173 of Cps4B (Fig. S4). Although this loop could be important in binding the phosphotyrosine, it does not confer any specificity of Cps4B to CpsCD, since our results show that Cps4B readily dephosphorylates Wzc from *E. coli* K-30 (Fig. 6).

## Conclusions

Bacterial tyrosine kinases, such as Wzc_K-30_, are involved in a plethora of processes ranging from production of CPS to regulation of virulence functions.^3^ The function of these proteins is crucially dependent on phosphatases, which are homologs of either Cps4B in Gram-positive bacteria or Wzb_K-30_ in Gram-negative bacteria. Due to the extent of the important processes that are regulated by these enzymes, they are of potential interest for drug development efforts. In this context, the solved structures may provide an important starting point for the development of new antibacterial drugs.

## Materials and Methods

### Protein production and purification

The Wzb_K-30_ and Cps4B proteins were produced and purified as previously described.^41^ An additional Wzb_K-30_ construct with cleavable His tag was prepared by amplifying the *wzb* gene from the original construct with PCR and then cloning it into the pEHisTEV vector.^42^ Expression and purification of this construct were essentially identical with those of the construct without the cleavable His tag.^41^ However, during purification, the His tag was removed using tobacco etch virus protease and a second Ni^2+^ affinity column was used to remove uncleaved protein and the protease. The Cps4B mutants Arg139Ala and Arg206Ala were prepared using a QuikChange Site-Directed Mutagenesis Kit (Stratagene) using the following oligonucleotides provided by Operon:

cps4b_R139A_fwd 5′-GAGCATCATACGCCTCAATGTGGGCAATG-3′,
cps4b_R139A_rev 5′-CATTGCCCACATTGAGGCGTATGATGCTC-3′,
cps4b_R206A_fwd 5′-CC ATATGAGGAGGCGCACCGTCTAGATTGTGC-3′ and
cps4b_R210A_rev 5′-GCACAATCTAGACGGTGCGCCTCCTCATATGG-3′.

Expression and purification of the mutants were identical with those of the wild-type protein.^41^ Full-length Wzc_K-30_ was prepared as described previously.^18^

### *In vitro* dephosphorylation of Wzc and Western blot analysis

To test whether Cps4B can dephosphorylate Wzc, we supplemented a 50-μl sample of purified Wzc (∼ 0.5 mg/ml in 50 mM Tris–Cl, pH 7.0, 500 mM NaCl and 0.008% DDM) with 30 μg of Cps4B or Wzb (positive control). The samples were incubated overnight at 4 °C, and the phosphorylation state of Wzc was checked by Western immunoblot analysis using an anti-phosphotyrosine antibody (SIGMA).

### *In vitro* activity assays

The activities of Cps4B, Cps4B R139A and Cps4B R206A were checked using the artificial substrate pNPP. The reactions contained 25 mM Tris–Cl, pH 8.0, 50 mM NaCl and 0.05 mg/ml of Cps4B and were started by addition of 2.5 mM pNPP. The metal dependency of the reaction was checked by adding 0.5 mM concentration of Cu^2+^, Mn^2+^, Co^2+^, Zn^2+^, Fe^2+^ or Ni^2+^. The reactions were performed at room temperature and were monitored by measuring the absorption at 405 nm.

### Structural biology

Wzb_K-30_ (with N-terminal His tag, crystal form I) crystallized in space group *P*3_2_21 as previously described.^41^ The structure was solved at 2.5-Å resolution by molecular replacement with the program PHASER^43^ and using the NMR structure of apo-Wzb (51% sequence identity; PDB ID 2FEK) from *E. coli* K-12^24^ as search model. Initial *F*_o_ − *F*_c_ electron density maps showed strong evidence for a phosphate ion bound to the active site, but refinement stalled (*R*/*R*_free_ of 30%/35%). Since twinning could be ruled out, we reintegrated the data and excluded any region that was affected by ice rings (MOSFLM).^44^ To exclude model bias introduced by the NMR structure as much as possible, we improved the initial phases from the molecular replacement step by density modification procedures implemented in PHENIX.AUTOBUILD^35^ and used the same program to build a completely new structure. Refinement of this structure with PHENIX.REFINE^35^ led to significantly improved *R*-factors of 22.0%/26.8% and good stereochemistry of the protein model.^45^

After removal of the His tag (see above), Wzb_K-30_ crystallized in space group *P*2_1_ (crystal form II) under a different condition: 0.1 M Tris–Cl, pH 8.5, 1.2 M Li_2_SO_4_ and 0.01 M NiCl_2_. The structure was solved by molecular replacement with PHASER^43^ using the previously refined model of the *P*3_2_21 crystal form as search model. Initially, the data set was indexed and processed (HKL2000^46^) in *P*2_1_2_1_2_1_. The structure could however only be refined (PHENIX.REFINE^35^) in *P*2_1_. The data collection and refinement statistics are listed in Table 1. In all structures, C13 (the active-site residue) adopts a disfavored Ramachandran conformation but appears well ordered. For the lower-resolution structure, two additional residues (K65 and S-1), both of which are disordered, are also in disfavored regions.

Cps4B from *S. pneumoniae* TIGR4 was crystallized in space group *P*4_3_2_1_2 as previously described.^41^ The structure was solved by single-wavelength (Cu-K, 1.54 Å) anomalous dispersion using a single samarium derivative crystal. A three-step protocol was used to prepare the heavy-atom derivative. In the first step, the crystal was soaked (10 min) in mother liquor supplemented with 50 mM samarium acetate. This step was followed by a quick back soak in mother liquor with 10% glycerol and a final soaking step with 20% glycerol. The derivative crystal was then flash frozen in liquid nitrogen prior to collection of a high-redundancy data set for structure solution (Table 1).

PHENIX.AUTOSOLVE^35^ was used to locate the single Sm site in the asymmetric unit of the crystal, allowing the phases to be calculated. The resulting experimental maps at 2.7-Å resolution were of excellent quality and yielded a complete model for Cps4B. Subsequent refinement of the structure with PHENIX.REFINE^35^ resulted in *R*/*R*_free_ factors of 17%/23%. A second orthorhombic crystal form of Cps4B (*P*2_1_2_1_2_1_)^41^ diffracted to beyond 1.7-Å resolution, allowing solution of a high-resolution crystal structure of Cps4B by molecular replacement, using the experimental 2.7-Å structure as search model (PHASER).^43^ The high-resolution structure was refined with PHENIX.REFINE^35^ to an *R*/*R*_free_ of 14%/19%. The data collection and refinement statistics are listed in Table 1. In the high-resolution structure, the metal ligands H136 and D13 adopt disfavored Ramachandran angles, but the electron density for both is clear.

The orthorhombic Cps4B crystals (*P*2_1_2_1_2_1_)^41^ were used for phosphate soaks. Three soaking solutions that contained mother liquor^41^ supplemented with increasing concentrations of phosphate [12.5 mM (solution I), 25 mM (solution II) and 50 mM (solution III)] were prepared. The crystal was quickly transferred from solutions I, II and III, after which each step was allowed to equilibrate for ∼ 5 min. Prior to data collection, the crystal was flash frozen in liquid nitrogen.

All structures were visualized and analyzed using Coot,^47^ and figures were prepared using PyMOL‡.

### Available supporting information

Figure S1 shows the superposition of the active-site regions of Wzb_K-30_ and Wzb_K-12_; Fig. S2, SSM-based alignment of Cps4B and structurally related phosphotriesterases; Fig. S3, anomalous difference Fourier map for metal ions in Cps4B; Fig. S4, alignment of Cps4B and homologs; and Fig. S5, phosphotyrosine residue modeled into the active site of Wzb_K-30_.

### Accession numbers

Coordinates and structure factors have been deposited in the PDB with accession numbers 2WJA, 2WJD, 2WJE and 2WJF.

## Figures and Tables

**Table 1:** Data collection and refinement statistics

	Wzb		Cps4B		
	Crystal form I PO_4_^3−^	Crystal form II SO_4_^2^^−^	Crystal form I Sm derivate	Crystal form II apo	Crystal form II PO_4_^3−^ complex
*Data collection*					
Space group	*P*3_2_21	*P*2_1_	*P*4_3_2_1_2	*P*2_1_2_1_2_1_	*P*2_1_2_1_2_1_
Crystal system	Trigonal	Monoclinic	Tetragonal	Orthorhombic	Orthorhombic
Unit-cell parameters					
*a* (Å)	90.0	101.9	88.9	44.0	44.0
*b* (Å)	90.0	53.1	88.9	58.5	58.7
*c* (Å)	83.5	113.9	92.3	114.8	115.2
α (°)	90	90	90	90	90
β (°)	90	90	90	90	90
γ (°)	120	90	90	90	90
Matthews coefficient (Å^3^ Da^− 1^)	2.94	2.26	3.21	2.60	2.60
Molecules per asymmetric unit	2	8	1	1	1
Resolution rage (Å)	45.0–2.5	50.0–2.2	30.0–2.8	30.0–1.9	30.0–2.2
Mosaicity (°)	0.6	1.0	0.2	0.1	0.733
Total observations	110,192	202,948	386,978	202,027	59,325
Unique reflections	12,623	59,571	17,311	40,850	15,250
Completeness (%)	90.8 (100)	96.9 (80.8)	99.3 (97.3)	92.5 (61.4)	98.8 (86.5)
*R*_merge_ (%)	7.5 (79.5)	8.7 (42.2)	5.5 (18.3)	1.9 (4.4)	9.0 (23.9)
Multiplicity	8.7	3.4 (2.7)	22.4	5.0	3.9
*I*/σ(*I*)	27.3 (5.4)	20.1 (3.2)	58.9 (18.2)	64.4 (27.8)	14.7 (4.4)

*Refinement*					
Resolution range (Å)	39.0–2.5	34.0–2.2	29.1–2.8	30.0–1.9	25.9–2.2
*R*/*R*_free_ (%)	22.0/26.8	21.2/26.2	17.3/23.0	13.9/19.0	15.8/22.4
rmsd bonds (Å)/angles (°)	0.002/0.570	0.005/0.856	0.006/0.973	0.009/1.106	0.007/0.946
Ramachandran plot (% allowed/disallowed)	98/2	99/1	99/1	99/1	99/1

Values in parentheses refer to the highest-resolution shell. The disallowed residues are discussed in Materials and Methods.

## Footnotes

‡ www.pymol.org
