# Supplementary material for: Crystal Structures of Wzb of Escherichia coli and CpsB of Streptococcus pneumoniae, Representatives of Two Families of Tyrosine Phosphatases that Regulate Capsule Assembly
Source: J Mol Biol. 2009 Sep 25;392(3):678–88. doi: 10.1016/j.jmb.2009.07.026 (PMC2777267; doi:10.1016/j.jmb.2009.07.026)

Crystal structures of Wzb of *Escherichia coli* and CpsB of *Streptococcus pneumoniae*, representatives of two different families of tyrosine phosphatases that regulate capsule assembly

Gregor Hagelueken[a,#], Hexian Huang[a,#], Iain Mainprize[b], Chris Whitfield[b], James H. Naismith[a,*]

[a]Centre for Biomolecular Sciences, The University of St. Andrews, Fife KY16 9RH, UK
[b]Department of Molecular and Cellular Biology, University of Guelph, Ontario N1G 2W1, Canada
[#]contributed equally
* E-mail: naismith@st-andrews.ac.uk,

SUPPORTING MATERIAL

Supplemental Figure 1 Active site close-up of Wzb_{K-30}. The NMR structure of apo-Wzb_{K-12} is superimposed (green) and shows the stabilization of the active site region by the phosphate ion.

Figure S1

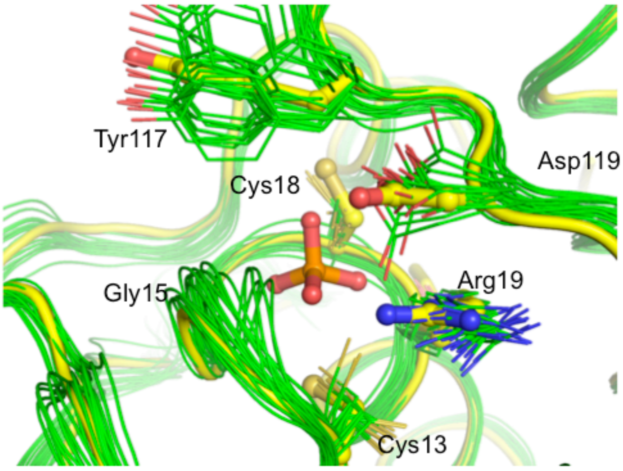

Supplemental Figure 2: SSM based alignment of Cps4B (color scheme as in Figure 3) with phosphotriesterases PHP from *E. coli* (PDB-id: 1BF6, blue) (Buchbinder et al., 1998)and SsoPox from *Sulfolobus solfataricus* (SsoPox, 2VC5, green) (Elias et al., 2008), showing the low structural similarity.

Figure S2

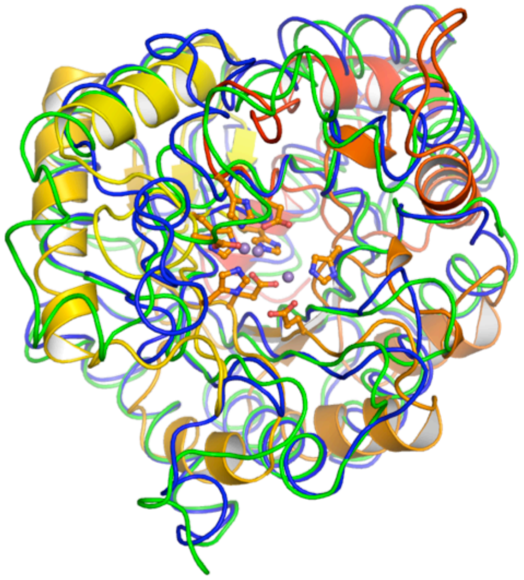

Supplemental Figure 3: Metal binding site of Cps4B. The purple mesh represents an anomalous difference Fourier map calculated from the native $P2_12_12_1$ apo-Cps4B dataset (Table 1) and is contoured at 12σ.

Figure S3

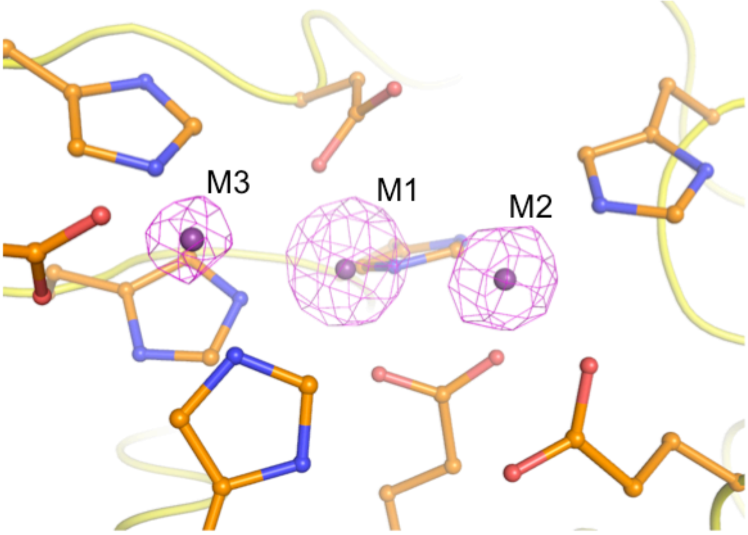

Supplemental Figure 4 Alignment of Cps4B with similar phosphatases from polysaccharide biosynthesis systems. Residues that are absolutely conserved are highlighted by purple boxes, and catalytically important residues Arg139 and Arg206 are highlighted in yellow. Secondary-structure elements are indicated by arrows (strands) and cylinders (helices). Metal ligands are identified by purple spheres. The position of the flexible FG(D/E)K/R loop is indicated by a grey bar.

Figure S4

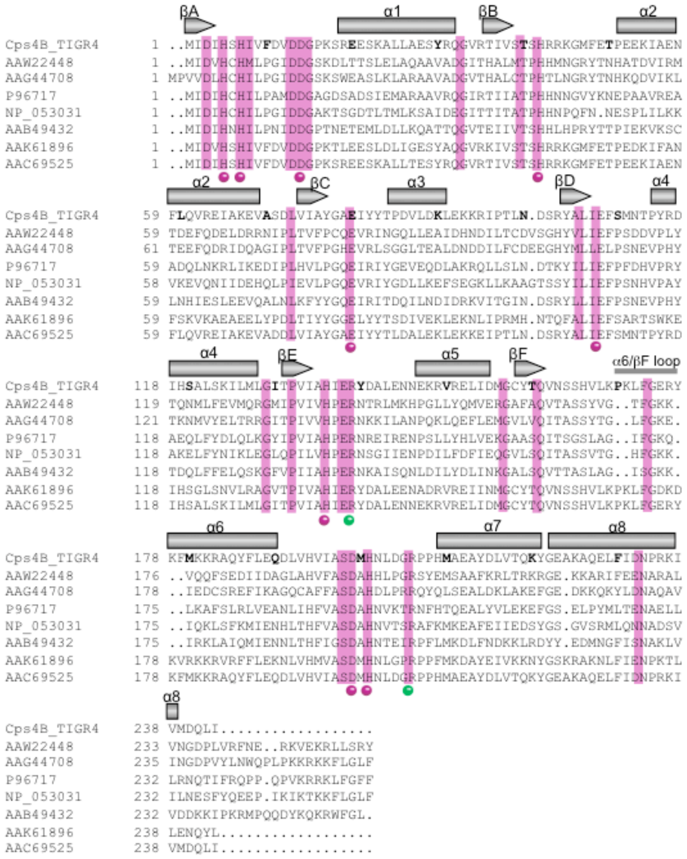

Supplemental Figure 5 Phosphotyrosine (pink) modeled onto the PO_4^{2-} ion (orange) which is bound to the active site of our Wzb_{K30} structure (yellow). This shows that Tyr119 can potentially stabilize the incoming substrate by a stacking interaction.

Figure S5

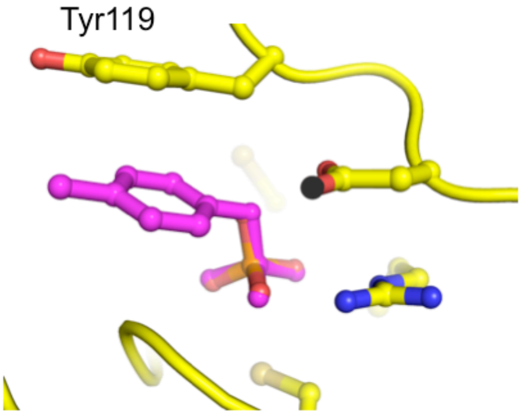

Supplement: Supplementary materials [file mmc1.pdf]
